# Monitoring of timely and delayed vaccinations: a nation-wide registry-based study of Norwegian children aged < 2 years

**DOI:** 10.1186/s12887-015-0487-4

**Published:** 2015-11-13

**Authors:** Øystein Rolandsen Riise, Ida Laake, Marianne Adeleide Riise Bergsaker, Hanne Nøkleby, Inger Lise Haugen, Jann Storsæter

**Affiliations:** Division of Infectious Disease Control, Department of Vaccines, Norwegian Institute of Public Health, P.O. Box 4404, Nydalen, NO-0403 Oslo, Norway; Division of Infectious Disease Control, Division Management, Norwegian Institute of Public Health, P.O. Box 4404, Nydalen, NO-0403 Oslo, Norway

**Keywords:** Vaccination coverage, Vaccination, Delay, Vaccination programme, Surveillance, Monitoring, Infant

## Abstract

**Background:**

Delayed vaccinations increase the risk for vaccine preventable diseases (VPDs). Monitoring of delayed vaccinations by using a national immunisation registry has not been studied in countries recommending a two-dose (3 and 5 months of age) primary series of e.g., pertussis vaccine. Surveillance/monitoring of all vaccinations may improve vaccination programmes functioning.

**Methods:**

We obtained information from the Norwegian immunisation registry (SYSVAK) on all programme vaccinations received at age up to 730 days in children born in 2010 (*n* = 63,382). Timely vaccinations were received up to 7 days after the recommended age. Vaccinations were considered delayed if they were received more than one month after the recommended age in the schedule.

**Results:**

In vaccinated children, timely administration of the subsequent three doses of pertussis and one dose of measles occurred in 73.8, 47.6, 53.6 and 43.5 % respectively. Delay for one or more programme vaccinations (diphtheria, tetanus, pertussis, polio, Haemophilus influenza type B, invasive pneumococcal disease, measles, mumps or rubella) was present in 28,336 (44.7 %) children. Among those who were delayed the mean duration was 139 days. The proportion of children that had vaccinations delayed differed among counties (range 37.4 %–57.8 %). Immigrant children were more frequently delayed 52.3 % vs. 43.1 %, RR 1.21 (95 % CI 1.19, 1.24). Children scheduled for vaccines in the summer holiday month (July) were more frequently delayed than others (1^st^ dose pertussis vaccine 6.5 % vs. 3.9 % RR 1.65 (95 % CI 1.48, 1.85). Priming against pertussis (2^nd^ dose), pneumococcal (2^nd^ dose) and measles (1^st^ dose) was delayed in 16.8, 18.6 and 29.3 % respectively.

**Conclusion:**

Vaccinations were frequently delayed. Delayed vaccinations differed among counties and occurred more frequently during the summer vacation (July) and in the immigrant population. Monitoring improves programme surveillance and may be used on an annual basis.

**Electronic supplementary material:**

The online version of this article (doi:10.1186/s12887-015-0487-4) contains supplementary material, which is available to authorized users.

## Background

The infant morbidity of pertussis and invasive pneumococcal disease (IPD) is high. Measles continues to be a risk for young children as long as the disease has not been eradicated. Vaccinations are essential in preventing diseases [1].

To achieve early protection the first dose of pertussis and pneumococcal vaccines is recommended at 6 weeks to 3 months of age followed by additional dose(s) for primary immunisation and a booster dose to maintain protection (i.e., 3 priming doses + 1 booster (3 + 1 schedule) or 2 priming doses + 1 booster (2 + 1 schedule)). The first dose of measles vaccine is recommended from 9 months of age or early in the 2^nd^ year of life [2, 3]. The variation in schedules among countries may depend on immunisation strategies and well-child services [2].

Vaccination coverage at age 2 years is often used to measure vaccination programme performance. However, it does not reflect whether children are appropriately protected at all times according to the national schedule. In Norway, vaccine preventable diseases (VPD) occur in children delayed for vaccinations [4, 5]. Results from a Swedish study indicate lower incidence of pertussis if the first vaccinations occurred on time [6]. The need for early and timely pertussis vaccination has recently been highlighted by the WHO [7].

Reports of considerable vaccination delay in Europe, Australia and the US have been published, but data are limited for a 2 + 1 schedule as used in Norway [8–10]. Monitoring of delay has been suggested [9]. Monitoring may identify obstacles and areas of improvement. Norway uses a national immunisation registry and reports high vaccination coverage at age 2 years (≥93 % national, ≥ 85 % county); however the proportion and duration of delayed vaccinations are unknown [11, 12]. Also, because a rota virus vaccine with age restriction was introduced in Norway in 2014, a system for monitoring timely immunisation is planned.

The aim of this study was to describe deviations from the Norwegian vaccination recommendations with a special focus on delayed vaccinations in children aged < 2 years. This was assessed by using the national immunisation registry that today is used for annual vaccination coverage publications.

## Methods

### The Norwegian infant vaccination programme

Until September 2014, the Norwegian vaccination programme for children aged ≤ 2 years included vaccines against 9 target diseases for all children plus 2 extra (BCG + Hepatitis B) for risk groups (Table 1). Vaccines against diphtheria (D), tetanus (T), pertussis (acellular(aP)), polio (IPV) and Haemophilus influenzae type b (Hib) are often administered in pentavalent combination vaccines with two doses at 3 and 5 months as priming and with an early booster at 11–12 months of age. A vaccine against invasive pneumococcal disease (IPD) is administered at the same ages. The first dose of vaccine against measles, mumps and rubella (MMR) is offered at 15 months of age. Hepatitis B vaccine is offered in early childhood where a parent originates from a non-low endemic hepatitis B country or if a family member is infected with hepatitis B. The Bacillus Calmette-Guérin (BCG) vaccine is recommended if a parent originates from a country with high prevalence of tuberculosis [13]. All Norwegian municipalities (*n* = 429) are obliged by law to provide well-baby clinic services. The services, including immunisations, follow national recommendations, but are flexible for each child. Well child visits are provided by nurses and doctors. Vaccinations are mainly provided by public health nurses. All services are voluntary and free of charge. The proportion of programme vaccinations provided outside the well-baby clinics is unknown, but is probably very small.

**Table 1:** The Norwegian Vaccination Programme during 2010–2012, aged < 2 years

Age (months)	Target disease	Vaccine	Number of injections
3	Diphtheria, tetanus, pertussis, polio, Haemophilus influenza type b	Infanrix-Polio + Hib® (GSK), Prevenar®^a^ (Pfizer)	2
	Invasive pneumococcal disease		
5	Diphtheria, tetanus, pertussis, polio, Haemophilus influenza type b	Infanrix-Polio + Hib® (GSK), Prevenar®^a^ (Pfizer)	2
	Invasive pneumococcal disease		
12	Diphtheria, tetanus, pertussis, polio, Haemophilus influenza type b	Infanrix-Polio + Hib® (GSK) Prevenar®^a^ (Pfizer)	2
	Invasive pneumococcal disease		
15	Measles,mumps,rubella	M-M-RVaxpro® (Sanofi Pasteur MSD)	1
From early age	Hepatitis B^b^	Engerix-B® (GSK)	varies
	Tuberculosis^b^	BCG-SSI®	1

^a^Change from Prevenar 7 to Prevenar 13, April 2011; ^b^Risk groups

### National immunisation registry

The Norwegian immunisation registry (SYSVAK) is a national registry that has been nationwide since 1995. The principal objective is to ensure that all children are offered adequate vaccination. It is mandatory for vaccinators to report all administered programme vaccines to SYSVAK. It is recommended to report previous doses e.g., vaccinations administered abroad. Notifications of vaccinations from the well-baby clinics are electronically transferred from the patient record systems to SYSVAK. Notifications on paper are also accepted. Information available in SYSVAK includes personal identification number, date of birth, sex, vaccine, date of vaccination, status of residency and municipality. Vaccination coverage at age 2 years is published annually [12].

### Study population

We extracted information from SYSVAK on all programme vaccinations received at age ≤ 730 days (up until 2 years of age) in children born in 2010. Data was collected May 23^rd^ 2013. We included children with permanent residency and a personal identification number that had at least one vaccination registered (*n* = 61,889). We examined vaccinations against 11 VPDs irrespective of the use of mono/combination vaccines or brand names. If more than one vaccination for the same target disease were recorded on the same day, this was regarded as a typing error and only one vaccination was counted. We also included vaccine doses administered earlier than the recommended age.

The number of children aged 2 years (residents with personal identification number) in the Norwegian Population Registry (NPR) as of December 31^st^ 2012 was *n* = 63,382 [11]. Thus, the number of children that did not receive any documented programme vaccines was 1493 (2.4 %).

### Definition of timely and delayed immunisations

We classified the recommended age to end at the greatest number of days that could equal the given number of months (3 months = 92 days, 5 months = 153 days, 12 months = 365 days and 15 months = 457 days). Timely vaccinations were calculated for vaccinated children receiving vaccinations ≤ 7 days after the recommended age. This was done to have a more precise estimate for when vaccines were administered according to schedule.

Delay was defined similarly to what was done by Luman et al. [8]. A vaccine was defined as delayed if it was received later than 1 month (31 days) after the recommended age. Delay was counted in days and started at age 124 days for vaccines due at 3 months, at age 185 days for vaccines due at 5 months, at age 397 days for vaccines due at 12 months and at age 489 days for vaccines due at 15 months. Delay was counted until receipt of the vaccine or until the child was 730 days, whichever came first. For each of the 9 target diseases we calculated the cumulative delay for all recommended doses, the number of days on which at least one dose was delayed. We did not count delay for the two additional vaccines that are only recommended to risk groups (Hepatitis B and BCG).

For example, suppose a child is given 1^st^ dose of pertussis vaccine (due at 3 months) at age 210 days, 2^nd^ dose (due at 5 months) at age 293 days and 3^rd^ dose (due at 12 months) at 518 days. The 1^st^ dose is delayed 87 days, the 2^nd^ dose is delayed 109 days and the 3^rd^ is delayed 122 days. The cumulative delay for pertussis vaccine is 292 days, since days for which both 1^st^ and 2^nd^ doses are delayed (26 days, ages 185 to 210) are only counted once. We included all doses, but due to Norwegian recommendations regarding protection [13], delay was not counted for 2^nd^ and 3^rd^ dose of Hib vaccine if the 1st dose was given at age ≥ 365 days. If pneumococcal vaccine 1^st^ dose was given ≥ 365 days, delay was not counted for the 3^rd^ dose.

Finally, we calculated delay for the complete series, defined as number of days of which at least one vaccine was delayed. Since delay for different vaccines may overlap, delay for the complete series may be smaller than the sum of delays for each individual vaccine. Delay was categorised as 0, 1–7, 8–30, 31–90, 91–180 or > 180 days.

### Analysis

Vaccination coverage was calculated as the number of children fully vaccinated at age ≤ 730 days divided by the number of resident children born in 2010 aged 2 years registered in the NPR as of December 31^st^ 2012 (national level, *n* = 63,382) [11]. In addition we calculated vaccination coverage for the first tree doses of pertussis and pneumococcal vaccinations. Similarly, the proportion of children with delayed vaccinations was found by dividing the number of children with delay by the population in the NPR. We presumed that children without any programme vaccines in SYSVAK were unvaccinated and thereby had maximum delay for all vaccines. Since we did not have any information on the children not registered in SYSVAK, they were not included when we assessed delay by sex or county of residence. Vaccinated children with unknown county of residence (*n* = 43) were excluded from the comparison among the 19 Norwegian counties. BCG vaccinated children were considered immigrants, as this vaccine is recommended only if a parent originates from a country with high prevalence of tuberculosis.

To study whether delay was more likely among immigrant children than others, we calculated risk ratios and risk differences from frequency tables with corresponding 95 % confidence intervals (CIs). As children resident in Norway may follow other International vaccination schedules deviating from the official Norwegian guidelines we examined the number of pertussis and hepatitis B doses administered in the immigrant and non-immigrant populations. We furthermore compared delay among children scheduled for vaccines in July and children scheduled for vaccination in any other month. Public services and kindergartens may have limited opening hours and services in July as this is the main summer vacation month in Norway. The scheduled month was based on month of birth. These analyses included only children registered in SYSVAK, since we did not have information on date of birth for those not registered in SYSVAK. All analyses were performed with STATA/SE 13.0 (StataCorp, College Station, Texas, USA).

The study was approved by the Regional Committee for Medical and Health Research Ethics, Southeast Norway. The committee found it acceptable that informed consent was not collected.

## Results and discussion

### Results

#### Timeliness of vaccinations

**Fig. 1:**
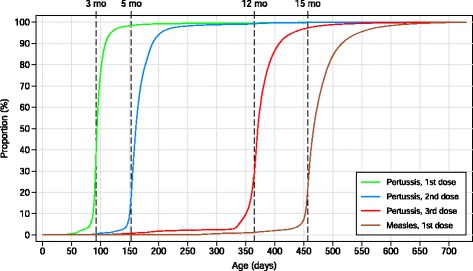
Cumulative distribution of age at vaccination. Pertussis: 1^st^ dose *n* = 61,703, 2^nd^ dose *n* = 61,316, 3^rd^ dose 60,153, measles: *n* = 59,070

Figure 1 shows the cumulative distribution of age at vaccination for pertussis and measles. The proportion of vaccinated children who were vaccinated ≤ 7 days after the recommended age was 73.8 % for the 1^st^ dose of pertussis vaccine, 47.6 % for the 2^nd^ dose of pertussis vaccine, 53.6 % for the 3^rd^ dose of pertussis vaccine and 43.5 % for measles vaccine. Few children received priming doses of pertussis (1^st^ and 2^nd^ dose) or the 1^st^ dose of measles earlier than the recommended age. Of the children who received pertussis vaccination, the proportion who received pneumococcal vaccine (given as a separate injection) on the same date was 97.1 % for the 1^st^ dose, 96.5 % for the 2^nd^ dose, and 96.2 % for the 3^rd^ dose. Results for pneumococcal vaccination were therefore similar to results for pertussis “Additional file 1”.

#### Delayed vaccinations

Delay for ≥ 1 programme vaccination was present in 28,336 of 63,382 (44.7 %) children (Table 2). Among those who were delayed the mean duration was 139 days (median 36 days). Of those who had vaccinations delayed, 7401 (26.1 %) were delayed ≥ 6 months. Vaccination coverage for VPDs was 93.1–95.7 % (complete programme 90.2 %).

**Table 2:** Vaccination delay and coverage during first 2 years of life among children born 2010, *n* = 63,382

	Children with delay		1–7 days		8–30 days		31–90 days		91–180 days		>180 days		If delayed, days			Vaccinated < 2 years	
	*n*	%	*n*	%	*n*	%	*n*	%	*n*	%	*n*	%	p25	p50	p75	*n*	%
Pertussis^a^	17,471	27.6	4025	6.4	5507	8.7	3115	4.9	1002	1.6	3822	6.0	8	25	116	60,153	94.9
1st dose	4070	6.4	640	1.0	724	1.1	548	0.9	112	0.2	2046	3.2	15	192	607	61,703	97.4
2nd dose	10,622	16.8	2923	4.6	3465	5.5	1278	2.0	369	0.6	2587	4.1	7	19	171	61,316	96.7
3rd dose (booster)	12,755	20.1	2265	3.6	3791	6.0	2538	4.0	677	1.1	3484	5.5	11	34	334	60,153	94.9
Pneumococcal^a,b^	18,674	29.5	4015	6.3	5529	8.7	3167	5.0	1080	1.7	4883	7.7	9	29	227	59,377	93.7
1st dose	5085	8.0	660	1.0	775	1.2	627	1.0	171	0.3	2852	4.5	23	294	607	61,119	96.4
2nd dose	11,775	18.6	2934	4.6	3519	5.6	1369	2.2	443	0.7	3510	5.5	8	23	309	60,652	95.7
3rd dose (booster)	13,912	21.9	2266	3.6	3802	6.0	2662	4.2	838	1.3	4344	6.9	12	42	334	59,156	93.3
Diphteria^a^	17,460	27.5	4024	6.5	5507	8.9	3117	5.0	1001	1.6	2318	3.7	8	25	116	60,162	94.9
Tetanus^a^	17,458	27.5	4024	6.3	5506	8.7	3119	4.9	1003	1.6	3806	6.0	8	25	116	60,174	94.9
Polio^a^	17,460	27.5	4021	6.3	5501	8.7	3114	4.9	1006	1.6	3818	6.0	8	25	116	60,148	94.9
Hib^a,c^	17,692	27.9	4015	6.3	5484	8.7	3102	4.9	1003	1.6	4088	6.4	9	26	134	60,614	95.6
Measles	18,492	29.2	3067	4.8	5511	8.7	4124	6.5	1203	1.9	4587	7.2	12	36	174	59,070	93.2
Mumps	18,573	29.3	3071	4.8	5517	8.7	4136	6.5	1215	1.9	4634	7.3	12	36	178	59,026	93.1
Rubella	18,565	29.3	3070	4.8	5517	8.7	4133	6.5	1214	1.9	4631	7.3	12	36	178	59,028	93.1
Complete series	28,336	44.7	5040	8.0	8072	12.7	5761	9.1	2070	3.3	7393	11.7	11	36	209	57,202	90.2

^a^Cummulative delay for the first 3 doses

^b^If pneumococcal vaccine 1st dose was given after age 365 days, delay was not counted for 3rd dose

^c^If Hib vaccine 1st dose was given after age 365 days, delay was not counted for the 2nd and 3rd dose

The proportion of children delayed for pertussis vaccination increased by subsequent doses and the cumulative delay was higher for the series of 3 doses than delay for the 3^rd^ dose (1^st^ dose 6.4 %, 2^nd^ dose 16.8 %, 3^rd^ dose 20.1 %, series of 3 doses 27.6 %). The proportion of children delayed for primary pneumococcal and measles vaccination was 18.6 and 29.2 %, respectively. Up to 3 months delay was more frequent for vaccination against measles than for the primary pertussis vaccination, 20.0 % vs. 12.1 %, respectively. Children delayed for pertussis, 1^st^ dose, were more likely to be delayed for measles vaccination compared to others, 70.8 % vs. 26.3 % RR 2.69 (95 % CI 2.63, 2.75).

**Fig. 2:**
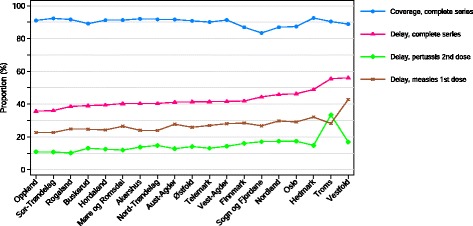
Vaccination coverage and delayed vaccinations by county in children aged < 2 years, born 2010, Norway, (n=61846). Legend: Complete series: Three doses: diphtheria, tetanus, pertussis, polio, haemophilus influenza type B (only 1 dose if 1st dose given at age ≥ 365 days), pneumococcal (only 2 doses if 1st dose given at age ≥ 365 days), 1 dose: measles, mumps, rubella vaccines. Vaccination coverage, proportion vaccinated at age 2 years. Delay: vaccination administered ≥ 1 month after due date

The proportion with delay did not differ by gender (boys 43.7 % vs. girls 43.0 % for the complete series). On county level, 37.4–57.8 % of children were delayed for ≥ 1 programme vaccination (median delay, range 23–42 days; Fig. 2). The two counties (Troms and Vestfold) with the highest proportion delayed vaccinations had vaccination coverage at 2 years ≥ 89 % for the complete series. When we compare the counties with highest (Vestfold) and lowest (Oppland) proportion of delayed vaccinations the immigrant population was 15.8 % vs 9.7 %, the proportion living in urban settlements was 85.3 % vs. 57.1 % and the proportion of adult higher education was 25.5 % vs. 21.5 % respectively [14].

#### Summer, delayed vaccinations

Children who according to month of birth were scheduled for vaccines in July were more often delayed than others: 1^st^ dose pertussis vaccine 6.5 % vs. 3.9 %, RR 1.65 (95 % CI 1.48, 1.85); 1^st^ dose pneumococcal vaccine 8.3 % vs. 4.4 %, RR 1.49 (95 % CI 1.35, 1.64); 1^st^ dose measles vaccine 40.8 % vs. 26.3 %, RR 1.56 (95 % CI 1.50, 1.61) (Table 3).

**Table 3:** Vaccinations scheduled in July vs. vaccinations scheduled all other months, *n* = 61,889

	Scheduled July	Scheduled all other months	RR (95 % CI)	RD (95 % CI)
Pertussis, delay				
1st dose, *n* (%)	335 (6.5)	2242 (3.9)	1.65 (1.48, 1.85)	2.6 (1.9, 3.3)
2nd dose, *n* (%)	877 (18.3)	8252 (14.5)	1.27 (1.19, 1.35)	3.8 (2.7, 5.0)
3rd dose, *n* (%)	1604 (28.6)	9658 (17.2)	1.67 (1.59, 1.74)	11.4 (10.2, 12.6)
Pneumococcal, delay				
1st dose	426 (8.3)	3166 (5.6)	1.49 (1.35, 1.64)	2.7 (1.9, 3.5)
2nd dose	972 (20.3)	9310 (16.3)	1.24 (1.17, 1.32)	4.0 (2.8, 5.2)
3rd dose	1676 (29.9)	10,743 (19.1)	1.56 (1.50, 1.63)	10.8 (9.5, 12.0)
Measles, delay				
1st dose, *n *(%)	2095 (40.8)	14,904 (26.3)	1.56 (1.50, 1.61)	14.6 (13.2, 16.0)

*RR* relative risk, *RD* risk difference (%), *CI* confidence interval

#### Immigrants, deviations and delayed vaccinations

Immigrant children were more delayed for the complete programme than others 52.3 % vs. 43.1 %, respectively, RR 1.21 (95 % CI 1.19, 1.24) and particularly for pneumococcal vaccination (38.7 % vs. 27.6 % RR 1.41 (95 % CI 1.37,1.44) (Table 4). Four or more doses pertussis vaccine and ≥ 3 doses hepatitis B vaccine were more frequently administered to immigrants than to non-immigrants 6.3 % vs. 1.1 % RR 5.7 (95 % CI 5.1,6.4), and 89.3 % vs. 2.9 % RR 30.5 (95 % CI 29.0,32.1) respectively.

**Table 4:** Vaccinations and delayed vaccinations in immigrants vs. nonimmigrants, born 2010, Norway, *n* = 63,382

	Immigrant^a^	Non-immigrant	RR (95 % CI)	RD (95 % CI)
	(*n* = 10,773)	(*n* = 52,609)		
Complete series^b^				
Vaccinated	9510 (88.3)	47,692 (90.7)	0.97 (0.97, 0.98)	−2.4 (−3.0, −1.7)
Delayed, *n *(%)	5638 (52.3)	22,698 (43.1)	1.21 (1.19, 1.24)	9.2 (8.2, 10.2)
If delayed, no of days, median (25p, 75 p)	50 (15, 242)	34 (11, 180)		
Pertussis				
≥ 3rd dose, *n* (%)	10,382 (96.4)	49,771 (94.6)	1.02 (1.01, 1.02)	1.8 (1.4, 2.2)
≥ 4doses, *n* (%)	680 (6.3)	579 (1.1)	5.7 (5.1, 6.4)	5.2 (4.7, 5.7)
Delayed, *n* (%)	3519 (32.7)	13,952 (26.5)	1.23 (1.19, 1.27)	6.1 (5.2, 7.1)
If delayed, no of days, median (25p, 75 p)	27 (9, 95)	14 (8, 128.5)		
Pneumococcal				
Vaccinated, *n* (%)	9958 (92.4)	49,419 (93.9)	0.98 (0.98, 0.99)	−1.5 (−2.0, −0.9)
Delayed, *n* (%)	4174 (38.7)	14,500 (27.6)	1.41 (1.37, 1.44)	11.1 (10.2, 12.2)
If delayed, no of days, median (25p, 75 p)	41 (11, 285)	26 (9, 195)		
Measles				
1st dose, *n* (%)	10,081 (93.6)	48,989 (93.1)	1.00 (1.00, 1.01)	0.5 (0.0, 0.9)
Delayed, *n* (%)	3221 (29.9)	15,271 (29.0)	1.11 (1.07, 1.15)	0.9 (0.0, 1.8)
If delayed no of days, median (25p, 75 p)	38 (13, 148)	35 (12, 186)		
Hepatitis B				
≥ 3rd dose, *n* (%) 3rd dosed dose, no (%)	9620 (89.3)	1540 (2.9)	30.5(29.0,-32.1)	86.4 (85.8, 87.0)

^a^Immigrants was defined as children registered with BCG vaccination

^b^Three doses of diphtheria, tetanus, pertussis, polio, haemophilus influenza type B (only 1 dose if 1^st^ dose given at age ≥ 365 days), pneumococcal (only 2 doses if 1^st^ dose given at age ≥ 365 days)and one dose of measles, mumps, rubella vaccines

#### Discussion

This study documents that 45 % of all children aged ≤ 730 days received at least one vaccination later than recommended. Vaccination coverage at age 2 years was acceptable and above 93 % for vaccines against 9 target diseases. Furthermore, the proportions that were delayed for the complete series differed among counties, whereas overall coverage did not differ among counties. Delayed vaccinations occurred more frequently during the main vacation month of July and in the immigrant population.

Delayed vaccinations in approximately 45 % of children for the complete series is similar to findings in a recent American survey by Glanz (49 %), but less than the 74 % found in a previous American survey by Luman [8, 15]. These two and our study used a similar definition for delayed vaccinations. This shows that although different schedules and data sources were used, delay for the complete series is a common phenomenon. Moreover, since a considerable proportion of children have their vaccines delayed, this should be a public health concern.

Most children in our study were delayed for a short period of time (median 36 days). Around 26 % of those delayed were delayed for ≥ 6 months. Due to the present Norwegian epidemiological situation, long delay for pertussis immunisation is a greater concern than long delay for measles vaccination. The duration of delay was longer in one American study [8], where the median delay was 126 days and 37 % had long delay.

Delayed priming against pertussis occurred in 17 %. This is less than what has been found in other countries including Flandern (Belgium) [9, 10, 16]. Other studies have consistently found that delay increases by number of doses [8–10]. Compared to a three dose priming schedule, the two dose priming schedule used in Norway and some other European countries has the benefit of less immunisation visits and thereby theoretically fewer options for delayed vaccinations. A Swedish study found that pertussis vaccination has a protective effect already after the first and second dose. It was hypothesised that a considerable proportion of pertussis could have been avoided if all children had been vaccinated on time [6].

We found that almost one in five children had the primary pneumococcal series (2^nd^ dose) delayed. These children may not receive the earliest doses when protection is needed [17] as full protection is presumably achieved 2 weeks after the 2^nd^ dose [18]. The incidence of vaccine type IPD has decreased substantially since the introduction of pneumococcal vaccines in the Norwegian programme [18]. However, IPD has been reported in some cases where the primary doses had been delayed [19, 20].

Delay for measles vaccination, 1^st^ dose, at 29 %, is in line with other studies ranging from 23 to 57 % [8–10, 21]. It has been shown that delay may reduce effective vaccine coverage in young age groups [21]. Delayed measles vaccinations keep children susceptible and increase the number of young children affected during outbreaks as observed in a recent outbreak in Oslo [4]. During this outbreak, vaccine failure was rare. Children who should have been vaccinated according to the schedule, transmitted measles to children who were too young to receive the vaccine according to the Norwegian programme. Hence, improving the proportion of children following the schedule could be more effective in protecting young children than recommending measles vaccine at an earlier age. The argument for timely measles vaccination is further supported by a recent study showing that delaying MMR vaccination increases the risk of MMR post vaccination seizures [22].

Examining delay for all vaccinations at national level only, may result in regional differences being overlooked (range 37–58 %). A wide variation within countries was also found in a study from low- and middle income countries [23]. Interestingly, the high proportion of children with delayed vaccines in some Norwegian counties was not reflected in the coverage estimates at age 2 years, so most parents had their children vaccinated. Long travel time to clinics has been suggested as a reason for delay [9]. Our study did not focus on geography or remoteness. However, delay was common both in the capital county and in one of the geographically smallest counties (Vestfold). Hence, a long distance to health care facilities is probably not a substantial reason for delay, suggesting other regional barriers such as organisational and local traditions [23].

The priming with pertussis vaccine (2 doses) and the 1^st^ dose with measles vaccine were considerably delayed where children were scheduled for vaccination in July compared to other months of the year. Data on delayed immunisations in the summer vacation season have to our knowledge not been presented before. Travelling is frequent during summer and this may easily spread measles and pertussis. To avoid susceptibility, parents should be informed of options of earlier vaccination at their first visits to well-baby clinics.

A higher proportion of children were delayed for vaccinations if they had immigrant parents. This was consistent with a survey from Flandern, where having a mother born outside the European Union was a risk factor for childhood vaccination delay [10]. Delay in minority groups has also been described elsewhere [8, 9]. Interestingly, we found a higher risk for delayed pneumococcal and pertussis vaccination than for delayed measles vaccination in immigrants compared to non-immigrants. As measles vaccination only requires one visit in children aged < 2 years, the number of visits may affect the risk of delay.

Strengths of this study include that our data represent the vaccination history of all children ≤ 2 years born in 2010 resident in Norway. We showed how data from a national immunisation registry used to publish annual coverage estimates could be analysed to improve surveillance by including data on timeliness and delay.

Limitations of our study include that information from SYSVAK was not linked with NPR. Therefore we do not have information about unvaccinated children. In addition, information from SYSVAK and the NPR was not obtained on the same date and both registries are regularly updated. We do not believe that this would have a considerable impact on our results. This assumption is supported by almost identical coverage estimates for individual vaccines compared to the official Norwegian coverage estimates [11].

Through SYSVAK, we do not know immigrant status, so our result must be interpreted with caution. However as 90 % of BCG vaccinated also had 3 doses hepatitis B vaccine (risk groups are overlapping, but not similar), BCG vaccination can be used as a proxy parameter to indicate immigrant children.

Until now, a delay in vaccination according to the programme results in unnecessarily unprotected children, especially for pertussis, that is an endemic disease, and for measles during outbreaks. A high vaccination coverage in young children and in the general population will ensure herd immunity and some protection for those who are not timely following the schedule or are too young to receive vaccinations. However, when introducing a vaccine with age restrictions, like rotavirus in Norway in autumn 2014, a delay can result in more non-vaccinated children since the child might be too old to be offered the vaccine.

Information focusing on early vaccine protection, more generous office hours and improved reminder systems, e.g. text messages or smart phone applications for parents, may improve timeliness of vaccinations. Every opportunity should be used to vaccinate and accelerated schedules and multiple vaccinations may be used to avoid further delay in children who have missed out of previous vaccination opportunities.

## Conclusions

Our results show that vaccinations were frequently delayed although vaccination coverage was high for vaccines targeted at specific diseases. Delay increase susceptibility for VPD, especially for delayed priming against pertussis, IPD and measles. Based on the present infectious disease burden in Norway, the first doses of pertussis vaccines should be administered on time, whereas a short delay for measles vaccination is more acceptable. However, children should not have their measles vaccinations delayed prior to the summer and vacation season due to travel and tourism. Knowledge about delayed vaccination can be used to improve information to vaccination providers and parents on the benefits of adhering to age recommendations, that until now may have been under-communicated. Monitoring of vaccination delay in addition to coverage is also a useful tool to improve programme surveillance. This is feasible in countries with a national vaccination registry. We would suggest monitoring of timely and delayed vaccinations also in the future.

## Additional file

Additional file 1:
**Cumulative distribution of age at vaccination. **1^st^ dose *n* = 61,119, 2^nd^ dose *n* = 60,652, 3^rd^ dose *n* = 59,156. (PDF 17 kb)

## Abbreviations

VPD: Vaccine preventable disease
IPD: Invasive pneumococcal disease
BCG: Bacillus Calmette-Guérin
SYSVAK: Norwegian immunisation registry
NPR: Norwegian Population Registry
CIs: Confidence intervals
Hib: Haemophilus influenzae type b
IPV: Inactivated polio vaccine
RRs: Risk ratios
RDs: Risk differences
