# Supplementary figures and images for: Monitoring of timely and delayed vaccinations: a nation-wide registry-based study of Norwegian children aged < 2 years
Source: BMC Pediatr. 2015 Nov 13;15:180. doi: 10.1186/s12887-015-0487-4 (PMC4643514; doi:10.1186/s12887-015-0487-4)

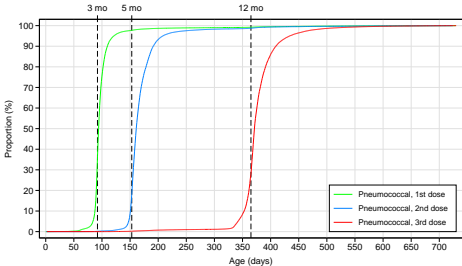

Supplement: Additional file 1: — Cumulative distribution of age at vaccination. 1st dose n = 61,119, 2nd dose n = 60,652, 3rd dose n = 59,156. (PDF 17 kb) [file 12887_2015_487_MOESM1_ESM.pdf]
